# A narrative review of digital biomarkers in the management of major depressive disorder and treatment-resistant forms

**DOI:** 10.3389/fpsyt.2023.1321345

**Published:** 2023-11-23

**Authors:** Annarita Vignapiano, Francesco Monaco, Claudio Pagano, Martina Piacente, Federica Farina, Gianvito Petrillo, Raffaella Sica, Alessandra Marenna, Jae Il Shin, Marco Solmi, Giulio Corrivetti

**Affiliations:** ^1^Department of Mental Health, Salerno, Italy; ^2^European Biomedical Research Institute of Salerno (EBRIS), Salerno, Italy; ^3^Dipartimento di Scienze Aziendali—Managment e Innovation Systems, Università di Salerno, Salerno, Italy; ^4^Innovation Tecnology e Sviluppo (I.T. Svil), Salerno, Italy; ^5^Department of Pediatrics, Yonsei University College of Medicine, Seoul, Republic of Korea; ^6^Severance Underwood Meta-Research Center, Institute of Convergence Science, Yonsei University, Seoul, Republic of Korea; ^7^Department of Psychiatry, University of Ottawa, Ontario, ON, Canada; ^8^On Track: The Champlain First Episode Psychosis Program, Department of Mental Health, The Ottawa Hospital, Ontario, ON, Canada; ^9^Clinical Epidemiology Program, Ottawa Hospital Research Institute, University of Ottawa, Ottawa, ON, Canada; ^10^School of Epidemiology and Public Health, Faculty of Medicine, University of Ottawa, Ottawa, ON, Canada; ^11^Department of Child and Adolescent Psychiatry, Charité—Universitätsmedizin, Berlin, Germany

**Keywords:** major depressive disorder, digital biomarkers, wearable devices, artificial intelligence, personalized treatment, mental healthcare

## Abstract

**Introduction:**

Depression is the leading cause of worldwide disability, until now only 3% of patients with major depressive disorder (MDD) experiences full recovery or remission. Different studies have tried to better understand MDD pathophysiology and its resistant forms (TRD), focusing on the identification of candidate biomarkers that would be able to reflect the patients’ state and the effects of therapy. Development of digital technologies can generate useful digital biomarkers in a real-world setting. This review aims to focus on the use of digital technologies measuring symptom severity and predicting treatment outcomes for individuals with mood disorders.

**Methods:**

Two databases (PubMed and APA PsycINFO) were searched to retrieve papers published from January 1, 2013, to July 30, 2023, on the use of digital devices in persons with MDD. All papers had to meet specific inclusion criteria, which resulted in the inclusion of 12 articles.

**Results:**

Research on digital biomarkers confronts four core aspects: (I) predicting diagnostic status, (II) assessing symptom severity and progression, (III) identifying treatment response and (IV) monitoring real-word and ecological validity. Different wearable technologies have been applied to collect physiological, activity/sleep, or subjective data to explore their relationships with depression.

**Discussion:**

Depression’s stable rates and high relapse risk necessitate innovative approaches. Wearable devices hold promise for continuous monitoring and data collection in real world setting.

**Conclusion:**

More studies are needed to translate these digital biomarkers into actionable interventions to improve depression diagnosis, monitoring and management. Future challenges will be the applications of wearable devices routinely in personalized medicine.

## Highlights

- Digital biomarkers show promise in predicting and assessing mood disorders.
- Smartphone data aids in tracking depression severity and treatment responses.
- Wearable devices enhance real-world monitoring of mood disorders.
- Artificial intelligence advances offer new diagnostic and therapeutic possibilities.
- Integration of technology improves major depressive disorder (MDD) diagnosis and personalized treatment.

## 1 Introduction

Globally, depression is estimated to affect 300 million individuals and is the leading cause of disability worldwide (1) and will become the leading cause of disability globally by 2030 (1). The prevalence of depressive disorders is highest among young adults aged 18 years (2–4). Onset during adolescence poses a particularly elevated risk of recurrence and long-term impairment in real-life functioning (5, 6). Despite increased accessibility of treatment over the last four decades prevalence rates have remained static (7). Even the best available treatments are largely unsuccessful at producing lasting outcomes, as approximately 40%–50% of patients relapse within 1–2 years of receiving treatment (8, 9). Symptoms of depression may manifest on multiple levels, including subjective emotional, cognitive, behavioral, and physical. In the depression field there is a strong need for monitoring clinical evolution and treatment responses in a more efficient manner to identify the treatment-resistant depression (TRD) forms. TRD is a condition characterized by persistent or recurrent depressive symptoms despite adequate treatment with one or more antidepressant medications (10). Approximately one-third of individuals with major depressive disorder (MDD) do not achieve full remission of symptoms even after trying two suitable trials of antidepressants without adequate response (11, 12). Despite its medical importance MDD is poorly defined and diagnosed since its diagnosis is mostly based on data subjectively reported by the patients themselves (13–15) and lack of objective, clinically relevant outcome measure. It is still debated whether mental disorders should be conceptualized as discrete entities (categorical approach) such as DSM or ICD or as phenomena along a continuum of severity (dimensional approach). The US National Institute of Mental Health proposed a new approach for research on mental disorders, the research domain criteria (RDoC) (16), a project aimed at re-orienting research on etiology and pathophysiology of psychopathological phenomena from category-based to dimension-based and at incorporating genetics, neuroimaging, and cognitive features into diagnostic schemes. The focus of research in mood disorders has shifted to more quantifiable metrics, while behavioral aspects have diminished markedly in importance (17). Digital biomarkers have significant value in psychiatric conditions like schizophrenia, autism, and PTSD. In schizophrenia, they assist in early diagnosis, symptom tracking, and treatment optimization, enhancing patient care. For autism, digital biomarkers are crucial for monitoring social interactions and enabling early diagnosis and personalized interventions. In PTSD, these biomarkers aid in monitoring physical and behavioral responses, supporting early intervention and symptom assessment. Digital biomarkers are defined as objective, quantifiable physiological and behavioral data that are collected and measured by means of digital devices such as portables, wearables, implantables, or ingestibles. Digital technologies offer promising tools for detecting MDD and depression-related symptoms objectively and precisely (18, 19). These technologies enable the remote collection of large volumes of clinically relevant data, which may be less burdensome than traditional in-clinic visits and more reflective of clinically relevant changes (20, 21). Wearable technologies, such as smartwatches and novel sensors, can generate valuable digital biomarkers of depression in real-world settings (22) building a digital phenotyping, defined as the moment-by-moment quantification of the individual-level human phenotypein its own environment using apps from smartphones or other personal devices (19, 23). In the recent years, several digital biomarkers have been investigated for MDD characterization and diagnosis such as measure patterns of physical activity (24, 25) features from voice samples (26, 27), light exposure measurements (25), mobile phone global positioning systems (GPS) and normal usage of smartphones such as usage duration and frequency (22).

## 2 Aims of this review

This review investigates how digital technologies, such as wearables and smartphone apps, are revolutionizing the assessment and treatment of major depressive disorder (MDD) and treatment-resistant depression (TRD). It anticipates that as digital mental health assessment advances and precision medicine is applied, the quality of life for individuals with MDD and TRD will improve. The review also identifies research gaps and recommends further investigation.

## 3 Methods

### 3.1 Search strategy and study eligibility criteria

To identify studies on the use of wearable devices in depression research, a literature search was performed to two major health-related databases: PubMed and APA PsycINFO, focusing on articles published from 1st of January 2013 to 30 July 2023. We searched for papers in which abstracts included the terms: Depression AND Device OR Depression AND digital tools OR Depression AND digital biomarkers OR Depression AND smartwatch OR Resistant depression AND digital biomarker OR Resistant depression AND digital biomarker OR Resistant depression AND actigraphy OR Resistant depression AND smartwatch.

Studies were chosen based on these inclusion criteria: randomized controlled trial, retrospective study, cohort study, open study, expert opinion, concerning conceptualization, diagnosis of major depressive disorder (MDD) according to DSM-5 or ICD-10, studies published in English, studies carried out in humans and studies published in journals indexed in Embase or Medline.

The exclusion criteria were meta-analysis, review, duplicates, comments, editorials, case reports/case series, theses, proceedings, letters, short surveys and notes, studies irrelevant for the topic, unavailable full-text and studies that do not meet inclusion criteria.

**Figure 1:**
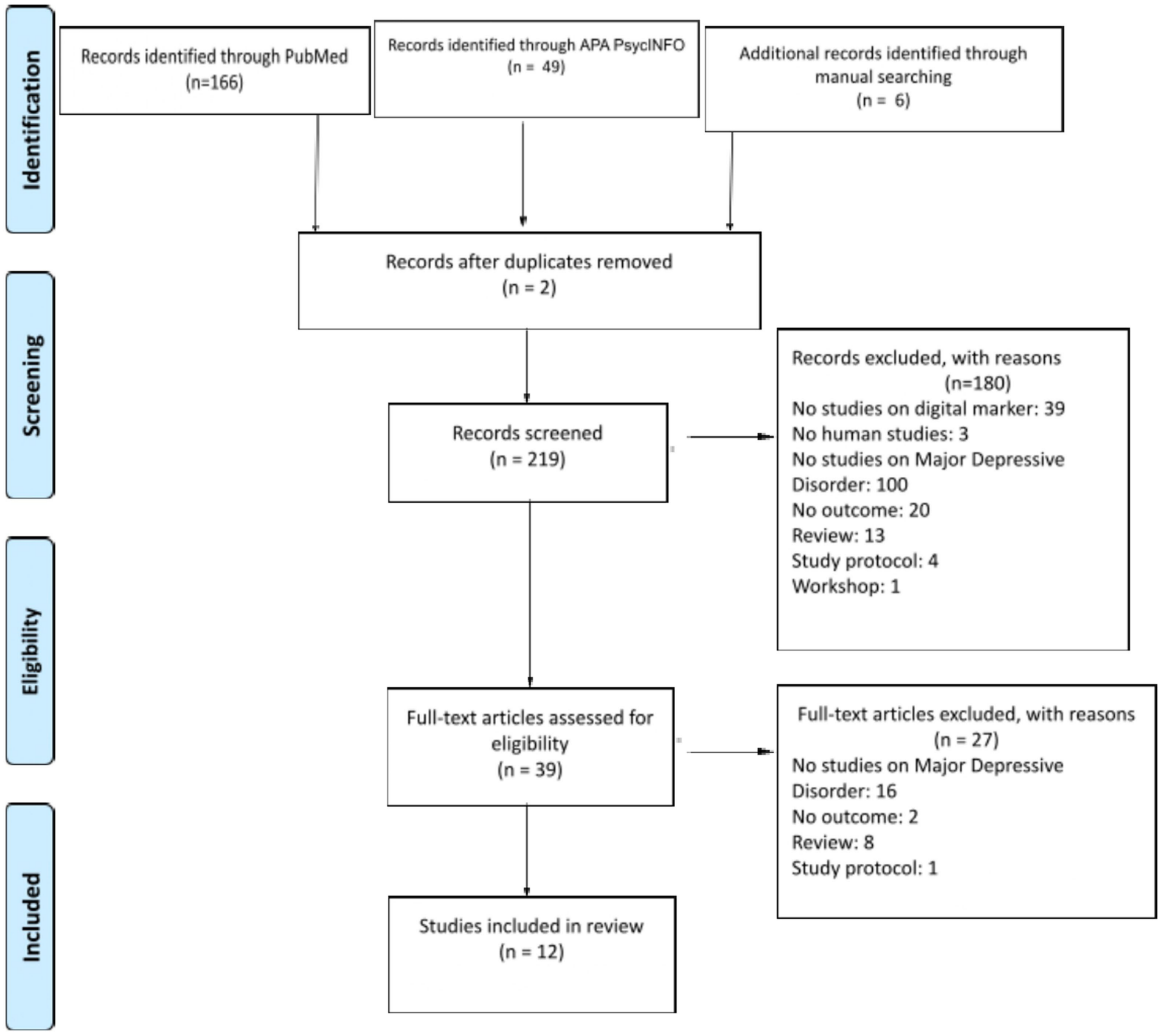
Flowchart of the study selection process. Adapted from Moher D, Liberati A, Tetzlaff J, Altman DG, PRISMA Group (2009). Preferred reporting items for systematic reviews and meta-analyses: the PRISMA statement. *PLoS Med*. 21. e1000097. doi: 10.1371/journal.pmed.1000097.

The PRISMA search process is presented in Figure 1.

We collected a total of 12 studies that made use of wearable devices to assess or monitor depressive symptoms and TRD or to predict MDD (Table 1).

**Table 1:** Studies using digital devices in subjects with MDD and TRD.

References	Subjects	Mood disorder diagnosis	Wearable device type	Device technology brands	Methods	Study experimentation duration	Mood assessed methods	Main points
Jacobson et al. (28)	23 patients (65% with primary MDD; 30% Bipolar II and 4% Bipolar I)	SCID-I	Wrist actigraph	Actiwatch	Actigraphs were worn at all times, except when bathing. The sampling frequency was 32 Hz and movements of ≥0.05 g were recorded. Voltage of movement was recorded for each minute	2 weeks	MADRS	Participants’ diagnostic group status can be predicted with a high degree of accuracy (predicted correctly 89% of the time)
Jacobson et al. (24)	15 MDD outpatients	MINI	Wrist actigraph	Actiwatch-L	Record of continuous movements (≥0.01 g) and ambient light exposure in lux every 15 min	1 week	BDI II; HAM-D	Passive movement and light data collected can be used to accurately assess both self-reported and clinician-rated depression severity
Siddi et al. (29)	510 MDD	LIDAS	Wrist-worn wearable device, and smartphone apps	Fitbit charge 2 and 3	HR was computed during the whole day (24 h) and just at night (from 00:00 to 05:59), as well as just during resting periods and during active periods separately. The average of each of the daily HR parameters was computed in the week before the PHQ-8 assessment across the follow-up	Up 2 years follow up	PHQ-8 (delivered through an app installed in an Android smartphone) every 2 weeks	During resting periods: decreases in HR variation during the day were related with an increased severity of depression. An HR at night was higher in participants with more severe depressive symptoms
Sverdlov et al. (30)	20 subjects with unipolar depression (MDD; PDD) 20 healthy controls	MINI	Smartphone apps	Android-based smartphone	During in-clinic visits three technologies were administered via mobile applications: an interactive tool for the self-assessment of mood, and a cognitive test; a passive behavioral monitor to assess social interactions and global mobility; a platform to perform voice recordings	2 weeks	HAM-D; MADRS	Correlation between various digital biomarker features and a clinical endpoint (MADRS total score) was assessed. Selected digital biomarker features (PHQ2 of Cambridge cognition; behavioral tracker features of BeHapp; neurophysiological features of Neurocart; EEG—resting state features of ElMindA Ltd. and EEG—BNA features of ElMindA Ltd.) were able to predict individual MADRS total scores, and use these models as classifiers
Abbas et al. (31)	18 MDD (11 women, 7 men)	MINI, MADRS	Smartphone apps		Video and audio captured during the smartphone assessment using the smartphone front-facing camera and microphone	4 weeks	Participants were asked by push-notification	Ability of digitally measured facial, vocal, and movement behaviors to measure depression severity and treatment response across 4 weeks of antidepressant treatment
Cormack et al. (32)	30 MDD (19 women, 11 men)	PHQ-9	Smartphone app, smartwatch	Apple iPhone, Apple Watch series 2	Cognitive and self-report assessments, heart rate and activity data	6 weeks	Complete the PHQ-8 every 2 weeks	High correspondence was observed between frequent assessments and established measures, showing moderate alignment between daily mood evaluations and validated depression questionnaires, and similar correlation for cognitive assessments with depression-sensitive tests
Kim et al. (33)	24 adolescents with MDD (17 girls, 7 boys), 10 HC	K-SAD -Present and Lifetime Version	Smartphone app		Smartphone usage time, physical movement distance, and the number of phone calls and text messages during the study period (STAR-DS app)	5 weeks	CDRS-R,CDI, BDI-II, C-SSRS CGI-S, CGAS, SCARED	Adolescents with MDD displayed higher call reception, possibly due to increased attention from family and friends. MDD participants exhibited extended smartphone usage, yet their usage wasn’t oriented towards social communication, marking a distinction from controls. MDD participants traveled longer distances than controls.
Zhang et al. (34)	316 MDD	PHQ-8	Smartphone NBDC		Passive and active remote monitoring technology apps and an activity tracker	2 years	PHQ-8	Increased time at home, inability to work or study, and diminished social interactions are reflected in the reduced amount of the NBDC sequence. Depression also may lead to misalignment of the circadian rhythm and make people’s life rhythms (such as sleep rhythms and social rhythms) more irregular
Mahendran et al. (35)	450 MDD	HAM-D	Smartwatch	Mi band—3	Gyroscope, accelerometer, heart rate monitor for recording the data from the gestures that the users make	1 week	—	The smartwatch data was used because it provided objective sensor data compared to the subjective questionnaire responses. After preprocessing and feature selection, logistic regression and random forest models were applied individually and then combined using a weighted average ensemble model. The results indicated that the weighted average ensemble performed better than the individual models, with random forest outperforming logistic regression
McNamara et al. (31)	60 MDE, 54 PC, 101 NCP	MINI for DSM-5	Sctigraphy		Daily physical activity, sleep consistency	1 week	MASQG, SHAPS	Psychiatric control groups can help to distinguish specific factors in the diagnosis of interest. Low positive emotionality is a strong differentiator of depression. Additionally, perceived sleep quality and impairment are also important predictors
Winkler et al. (36)	14 TRD	SCID-1	Actigraph	Actiwatch plus	Activity levels were measured with wrist actigraphy before and after ECT	4.1 ± 4.7 days of actigraphic measurement before ECT and 3.6 ± 2.1 days after ECT	HAM-D	Increase in light activity and circadian amplitude in patients with remission after ECT
Nishida et al. (37)	14 patients with medication resistant MDD	MINI	Actigraph	FS-750	Patients were instructed to wear the FS-750 system for a period of 7 days before the initiation of rTMS treatment and until rTMS treatment was completed	Actigraphic data were evaluated at baseline and in the first (rTMS sessions 1–3), second (rTMS sessions 4–7), and third (rTMS sessions 8–10) sections	HAM-D; PSQI	Sleep variables assessed by actigraphy did not show significant changes. A daytime physical activity response to rTMS occurred in early sessions

BDI-II, Beck Depression Inventory II; CDI, Children’s Depression Inventory; CDRS-R, Children’s Depression Rating Scale—Revised; CGAS, Children’s Global Assessment Scale; CGI-S, Clinical Global Impressions-Severity; C-SSRS, Columbia Suicide Severity Rating Scale; ECT, Electroconvulsive Therapy; EEG-BN, Electroencephalography Brain Network; HAM-D, Hamilton Depression Rating Scale; HR, Heart Rate; K-SADS, Kiddie Schedule for Affective Disorders and Schizophrenia for School-Age Children-Present and Lifetime Version; LIDAS, Lifetime Depression Assessment Self-Report; MASQ-GD, Mood and Anxiety Symptom Questionnaire—Short Form general distress subscale; MADRS, Montgomery-Asberg Depression Rating Scale; MDD, Major Depressive Disorder; MDE, Major Depressive Episode; MINi, Mini—International Neuropsychiatric Interview; NBDC, Nearby Bluetooth Device Count; NCP, No Current Psychopathology; PC, Psychiatric Control; PDD, Persistent Depressive Disorder; PHQ-2, Patient Health Questionnaire-2; PHQ-8, Patient Health Questionnaire-8; PSQI, Pittsburgh Sleep Quality Index; rTMS, Repetitive transcranial magnetic stimulation; SCARED, Screen for Child Anxiety Related Emotional Disorders; SCID-I, Structured Clinical Interview; SHAPS, Snaith-Hamilton Pleasure Scale; STAR-D, Sequenced Treatment Alternatives to Relieve Depression.

### 3.2 Study selection

The selection of studies for this review occurred in a two-stage process. Initially, two independent reviewers assessed the titles and abstracts of all the retrieved papers. In the subsequent stage, these same reviewers individually examined the full texts of the papers identified in the first phase. Any discrepancies between the two reviewers were resolved by involving a third reviewer.

### 3.3 Data extraction and data synthesis

Data extraction for each included study was carried out by two independent researchers, namely AV and FM, utilizing a standardized data extraction sheet in Microsoft Excel. The focus of this extraction encompassed several key subjects, including study design, participant characteristics, diagnosis of MDD, and digital device details derived from the original research.

In the event of any disparities in data entry between these two researchers, such discrepancies were thoroughly deliberated with two additional independent reviewers when deemed necessary.

## 4 Results

### 4.1 Literature search

The search across PubMed and APA PsycINFO bibliographic database produced a total of 215 records. Additionally, by conducting backward reference list checking and forward reference list checking, we discovered 6 new studies. After initial screening based on their titles and abstracts, 180 records were excluded. Out of the initial 39 references, the synthesis now comprises a total of 12 articles.

### 4.2 General description of included studies

Among the various studies examining wearable devices for individuals with MDD, approximately one- third employed actigraph units, while the rest utilized commercial wearable devices not originally designed for medical purposes. These included devices such as the Fitbit^®^ (Fitbit^®^, Inc., San Francisco, CA, United States), the Apple Watch (Apple Inc., Cupertino, CA, United States), and the Mi Smartwatch (Xiaomi Corporation, China). In addition, some studies relied on mobile applications (Apps).

Through a narrative synthesis of various reviews and in agreement with several authors’ perspectives, we have pinpointed specific domains where the integration of tools and digital markers significantly enhances clinicians’ capabilities in predicting, diagnosing, and providing care and treatment for individuals grappling with MDD and TRD.

The reviewed studies focused on gathering specific physiological, activity/sleep, or subjective data from individuals through digital devices, with the aim of exploring the relationship between these parameters and depression.

#### 4.2.1 Predictive modeling of diagnostic status

Multiple studies have showcased the promise of digital biomarkers, encompassing factors like movement intensity, light exposure, and smartphone usage patterns, in forecasting the diagnostic status of individuals grappling with mood disorders (19, 22, 38). Jacobson et al. (28) conducted a study to identify digital biomarkers for MDD and bipolar disorder (BD) and track symptom changes over 2 weeks. By analyzing movement patterns, they used extreme gradient boosting to achieve an 89% accuracy in predicting diagnostic groups and monitoring symptom changes. Combining MDD and BD data revealed potential transdiagnostic traits. Movement and light data were found relevant for detecting mood disorders and correlated with behaviors like energy levels, psychomotor activity, and sleep disturbances during mood episodes. Two studies focus on data analysis to identify important predictors in different contexts (35, 39). In their study Mahendran et al. (35), researchers used cardiac monitoring data, which included questionnaire responses and smartwatch sensor data. They then trained machine learning models such as logistic regression and random forest, and the results showed that the ensemble of models performed better than individual implementations, with random forest standing out. McNamara et al. (39), used a wide range of data, including demographic data, biobehavioral measurements, and self-report questionnaires to identify predictors of depression. The results revealed that psychosocial predictors such as negative self-referential thinking, rumination, self- reported sleep quality, and functional distress were important in predicting depression.

#### 4.2.2 Assessment of symptom severity and progression

Digital phenotyping has emerged as a valuable tool for evaluating the severity and trajectory of mood disorder symptoms. In the realm of assessing depression severity and treatment response in individuals with MDD, researchers have explored the utilization of digital biomarkers and technologies. Four notable studies shed light on this area: in the study conducted by Jacobson et al. (24), passive movement and light exposure data were analyzed in 15 medicated outpatients with MDD over a week. The study demonstrated that passive movement and light data could effectively gauge depression severity, even in cases of high severity. However, while modern lifestyles often drive the development of technology tailored for personal fitness, such as Fitbit^®^ and various apps that monitor vital signs like heart rate and body temperature, many devices used in research are repurposed for the advancement of mental health applications. Abbas et al. (31), used the AiCure smartphone app to track digital biomarkers associated with MDD under antidepressant therapy (ADT). These markers included voice, facial expressions, and movement indicators. The study found that monoamine ADTs, such as SSRIs and SNRIs, had a significant impact on digital biomarker with a reduction in symptom severity as assessed by the MADRS evaluation, indicating an improvement in motor functioning and a decrease in depression severity due to these treatments. Indeed, in Sverdlov et al. (30), the authors points out that common efficacy scales like HAM-D and MADRS are subjective and prone to bias. Digital technologies offer objective tools for depression symptom detection. Mobile apps and wearables can generate digital biomarkers in real-world settings. The study assessed seven digital technologies in individuals with unipolar depression and healthy controls, aiming to distinguish between them, build accurate classifiers, and explain variation in MADRS scores. Technologies were evaluated to identify digital biomarkers revealing correlations between different digital Importantly, selected digital biomarker features demonstrated predictive capabilities for individual. Therefore, Siddi et al. (29) in their study with up to 2 years follow up, based on data from the RADAR-MDD study involving 600 individuals with MDD, examined the relationship between heart rate (HR) parameters using a Fitbit^®^ device and the severity of depression. The findings showed that individuals with higher depression severity tended to have a lower resting HR variation throughout the day, and this association remained significant even after accounting for individual characteristics. The research indicates that passive behaviors, which are indicative of depression, are more common in individuals with greater depression severity, particularly during the nighttime when HR may be elevated due to the sleep problems often seen in those with MDD.

#### 4.2.3 Treatment response monitoring

Assessing treatment responses in the context of mood disorders constitutes a pivotal research domain. Abbas et al. (31) showcased the capacity of digital biomarkers, encompassing motor function, to accurately monitor shifts in depression severity throughout the course of antidepressant therapy. In a parallel attempt, Kim et al. (33) used smartphone data to prognosticate treatment outcomes among adolescents grappling with MDD, shedding light on the prospect of personalized treatment strategies through the avenue of digital phenotyping. In their research, Winkler et al. (36) investigated the impact of electroconvulsive therapy (ECT) on rest-activity patterns in patients with TRD. They studied 15 individuals with TRD who received ECT and used wrist actigraphy to measure their activity levels before and after treatment. They observed that Individuals who reached remission experienced notable enhancements in light activity, overall activity, and circadian amplitude and ECT had a limited impact on the timing of peak activity or actigraphic sleep measurements. In 2016, Nishida et al. (37) conducted an open-label pilot study on 14 medication-resistant MDD patients to assess the impact of rTMS on their rest-activity cycle and sleep disturbances. They administered 10 rTMS sessions targeting the bilateral dorsolateral prefrontal cortex and used waist actigraphy to measure changes in the rest- activity cycle. The results showed significant improvements in depression symptoms and sleep quality measured by rating scales, but actigraphy-based sleep measures did not exhibit substantial changes. Digital therapeutics are under study and represent a potential future clinical vista in this population (18). These findings, coupled with advancements in the realm of digital biomarkers and the refinement of neurostimulation parameters, hold potential for improving overall health results and the cost efficiency of MDD and TRD treatment.

#### 4.2.4 Real-world monitoring and ecological validity

Incorporating wearable devices into depression research offers several benefits. Wearable technology allows for ongoing and unbiased observation of individuals in their everyday environments. This enables the objective tracking of real-time changes and enhances the precision of monitoring treatment outcomes. Cormack et al. (32), involving individuals with MDD, explored the use of wearable technology for high-frequency cognitive and mood assessments over 6 weeks. The study found that daily assessments were practical and showed meaningful correlations with established measures of mood and cognition. While there was some improvement in mood, it varied among participants, highlighting the complexity of depression. In Kim et al. (33) paper, data from a smartphone app called “STAR-DS” was used to predict depressive symptoms and treatment responses in adolescents. The study found that call-related features, smartphone usage duration, and movement distance were important predictors of MDD. Call duration was especially significant in predicting treatment responses. Adolescents with MDD had different smartphone usage patterns compared to controls. This study emphasized the potential of smartphone behaviors in forecasting depression outcomes. Remote measurement technologies were used to monitor individuals with MDD in real-world settings. The study of Zhang et al. (34), found that Bluetooth device count (NBDC) data was correlated with depressive symptoms. Lower PHQ-8 scores were associated with increased social activities. Changes in NBDC data were linked to fluctuations in depressive manifestations and behaviors, including reduced social engagement, impaired work, or study performance, and disrupted circadian rhythms. This research highlighted the feasibility of using NBDC for monitoring individuals with MDD in real-life contexts.

## 5 Discussion

The review of the selected studies on predictive modeling, assessment of symptom severity and progression, treatment response monitoring, and real-world monitoring with ecological validity showcases the remarkable potential of digital biomarkers and technologies in advancing our understanding and management of MDD and TRD. The ability to predict diagnostic status with a high degree of accuracy using digital biomarkers is may be a transformative breakthrough. The integration of movement, light exposure, and smartphone data has not only enabled accurate predictions but has also revealed common features across mood disorders, highlighting the existence of transdiagnostic traits. This is in line with the research domain criteria (RDoC) criteria and opens new prospective for understanding the underlying mechanisms of mood disorders (35, 39). Assessing symptom severity and progression may be significantly enhanced by digital phenotyping, although this kind of technology is rapidly improving and the validity of these measurements need more strong confirmations. The use of passive movement and light exposure data, often repurposed from personal fitness technology, demonstrates the adaptability and versatility of digital biomarkers in assessing depression severity. Moreover, the impact of antidepressant therapies on digital biomarkers related to motor function may provides valuable insights into the mechanisms of action of these treatments. The ability to predict individual depression severity scores are groundbreaking advancements in the field, digital biomarkers potential role in predicting individual depression severity may be a groundbreaking advancement, once confirmed and replicated in larger populations studies, providing clinicians with even more personalized assessment instruments (29, 30). Monitoring treatment responses, especially in cases of treatment-resistant depression (TRD), is crucial for improving patient outcomes. These advancements, coupled with the refinement of neurostimulation parameters, hold the potential to enhance overall health outcomes and the cost-effectiveness of TRD care. This suggests that approaches, such as electroconvulsive therapy (ECT) and repetitive transcranial magnetic stimulation (rTMS), have the potential to positively impact the rest-activity cycle in TRD patients who achieve remission. The demonstrated effectiveness of digital biomarkers, including motor function, in tracking shifts in depression severity during treatment is a significant step forward (33, 36). Additionally, the potential for personalized treatment strategies through smartphone data analysis among adolescents with MDD holds promise for tailoring interventions to individual needs. Real-world monitoring with ecological validity using wearable technology and smartphone apps represents a paradigm shift in depression research. The ability to conduct daily assessments in natural environments provides a more comprehensive understanding of mood fluctuations and cognitive changes. Smartphone behaviors and Bluetooth device count data offer exciting prospects for predicting MDD and treatment responses. These findings underscore the dynamic nature of depression and the importance of considering real-world factors in assessment and treatment planning (33, 34).

## 6 Conclusion

This narrative review highlights the diverse research areas that underscore the versatility and potential of digital biomarkers and technologies in the diagnosis, assessment, and treatment of mood disorders. In this review, we delve into the technologies, available research findings, and implementation challenges most pertinent to the integration of digital psychiatry within MDD and its resistant forms. The integration of wearable devices with smart devices, such as mobile phones, has gained widespread acceptance due to their convenience and style (20). Notably, our review is the first in the literature to focus on wearable devices targeting depression assessed using DSM-5 or ICD-10 criteria. Although the different wearable device technologies were examined, the review falls short of reporting the effectiveness measure values, and therefore does not assess performance. This review underscores the potential for remote diagnosis and prediction using these devices. Future trends are anticipated with the emergence of new wearable devices that will introduce innovative diagnostic and therapeutic approaches like motion capture, speech analysis, and portable light therapy. These developments hold the promise of fundamental changes in the diagnosis and treatment of depression, potentially enabling early and precise diagnosis, personalized treatment for depression patients, and preventive measures for at-risk groups (40, 41). Digital psychiatry encompasses various aspects of healthcare, including delivery, illness surveillance, disease management, and treatment. Advances in artificial intelligence and machine learning are expected to serve as a crucial bridge for translating new data into clinically relevant digital biomarkers (22, 38).

Wearable devices are poised to play a critical role in medicine, particularly in the context of personalized telemedicine. Future research endeavors should continue to explore these areas, enhancing the precision and efficacy of digital phenotyping in mental healthcare, ultimately leading to an improved quality of life for individuals affected by MDD.

## Author contributions

AV: Conceptualization, Data curation, Methodology, Project administration, Resources, Writing – original draft. FM: Conceptualization, Methodology, Project administration, Writing – original draft. CP: Writing – review & editing. MP: Writing – review & editing. FF: Writing – review & editing. GP: Writing – review & editing. RS: Writing – review & editing. AM: Writing – review & editing. JS: Writing – review & editing. MS: Writing – review & editing. GC: Writing – review & editing.
